# Malignant Transformation of Plexiform Neurofibroma Due to Neglected Giant Soft Tissue Swelling of the Back: A Case Report

**DOI:** 10.7759/cureus.63807

**Published:** 2024-07-04

**Authors:** Wan Muhamad Amir Bin Abdul Halim, Samsuddin Bin Mat Hassan, Mazlina Bt Awang, Mohammad Anwar Hau Abdullah

**Affiliations:** 1 Orthopedics and Traumatology, Hospital Canselor Tuanku Muhriz UKM, Kuala Lumpur, MYS; 2 Orthopedics and Traumatology, Hospital Raja Perempuan Zainab II, Kelantan, MYS

**Keywords:** soft tissue tumours, malignant transformation, neurofibrosarcoma, malignant peripheral nerve sheath tumor, neurofibromatosis

## Abstract

Neurofibromatosis type 1 can be severe and associated with malignant transformation. Proper follow-up and monitoring are very important in preventing the malignant transformation of neurofibromatosis. We encountered a case of malignant transformation of plexiform neurofibroma into neurofibrosarcoma (also known as malignant peripheral nerve sheath tumor). She had been presenting with a large mass on her back for a few years, which was also associated with an ulcer. She underwent a wide-excision biopsy of her back, and the histopathology examination (HPE) came back with a malignant peripheral nerve sheath tumor. This case concludes that any patient with a known case of neurofibromatosis should undergo follow-up to detect any malignant transformation of the disease. Early detection of the malignant transformation of neurofibromatosis can help prevent the disease's progression. The main treatment is surgical resection; however, the risk of local recurrence is higher, especially in patients with neurofibromatosis type 1.

## Introduction

Neurofibromatosis (NF) is a disorder characterized by the formation of multiple cutaneous skin lesions involving the whole body. This condition is inherited via an autosomal dominant pattern. NF can be divided into two subtypes: NF type 1 (NF1) and NF type 2 (NF2). Both of the subtypes can be differentiated by different characteristics. Patients with NF1 may present with multiple cutaneous neurofibromas, cafe'-au-lait spots, plexiform tumors, Lish nodules, axillary or inguinal freckling, and optic gliomas, while those with NF2 are characterized by bilateral vestibular schwannomas and central nervous system (CNS) tumors such as meningiomas and ependymomas. There is no specific treatment for NF, and the main treatments for this condition include continuous monitoring and medical intervention when appropriate. People with neurofibroma develop generalized body neurocutaneous lesions. Delayed diagnosis and follow-up in patients with NF may lead to the malignant transformation of NF. Plexiform neurofibromas (PN) can undergo malignant transformation into neurofibrosarcomas (also known as malignant peripheral nerve sheath tumors, malignant schwannomas, or neurogenic sarcomas). As the neurocutaneous lesion underwent a malignant transformation, it began to grow larger and larger. When the lesion starts to grow, the tumor cells will infiltrate the surrounding mass, which may compromise and damage its blood supply. The surrounding tissue and skin undergo ischemic changes, leading to their eventual death (necrotic changes). Thus, the tumor starts ulcerating and becomes a fungating mass [1]. Experts estimate the lifetime risk of malignant transformation to be 4.6% [2]. According to studies, people with cutaneous neurofibroma have a threefold increased risk of developing malignant peripheral nerve sheath tumor (MPNST) [3-5]. We would like to report a case of malignant transformation of plexiform NF with fungating a mass on the back.

## Case presentation

**Figure 1:**
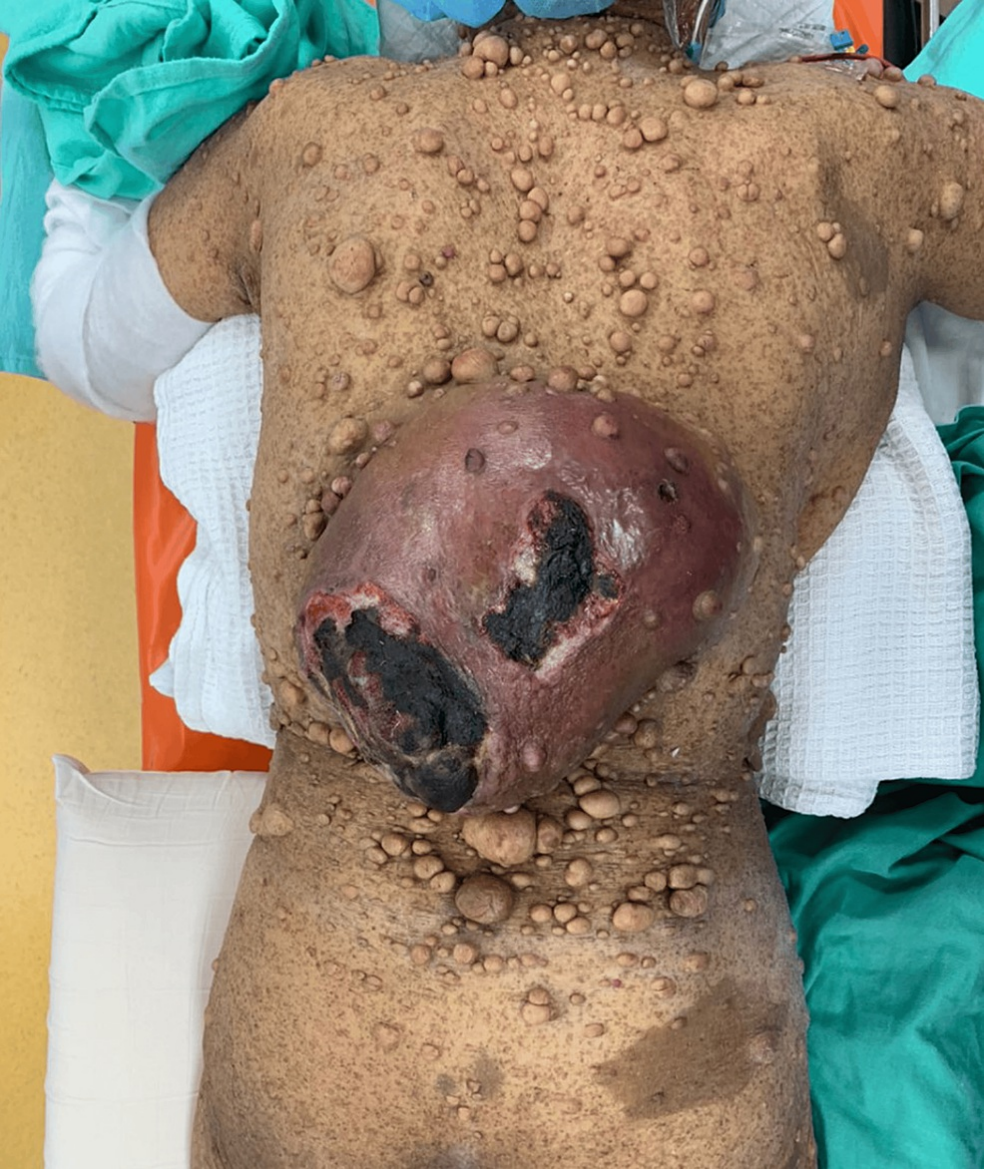
Huge swelling with ulcers on the patient's back.

**Figure 2:**
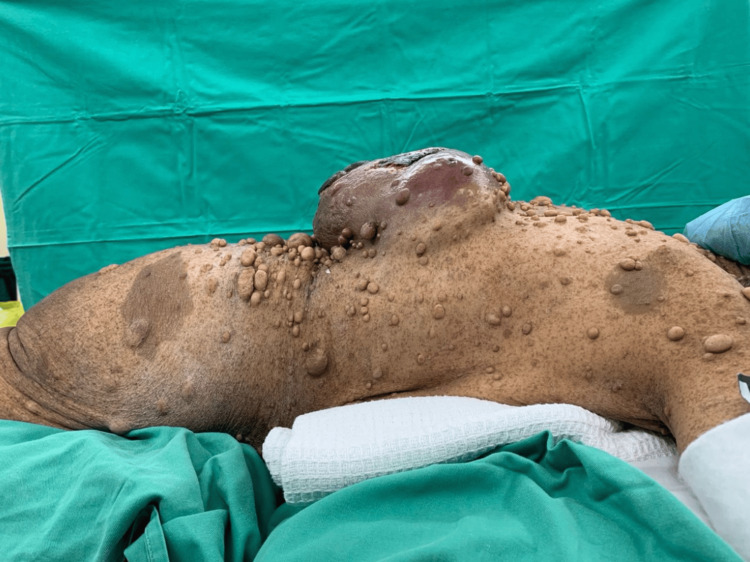
Clinical picture showing huge swelling on the patient's back.

**Figure 3:**
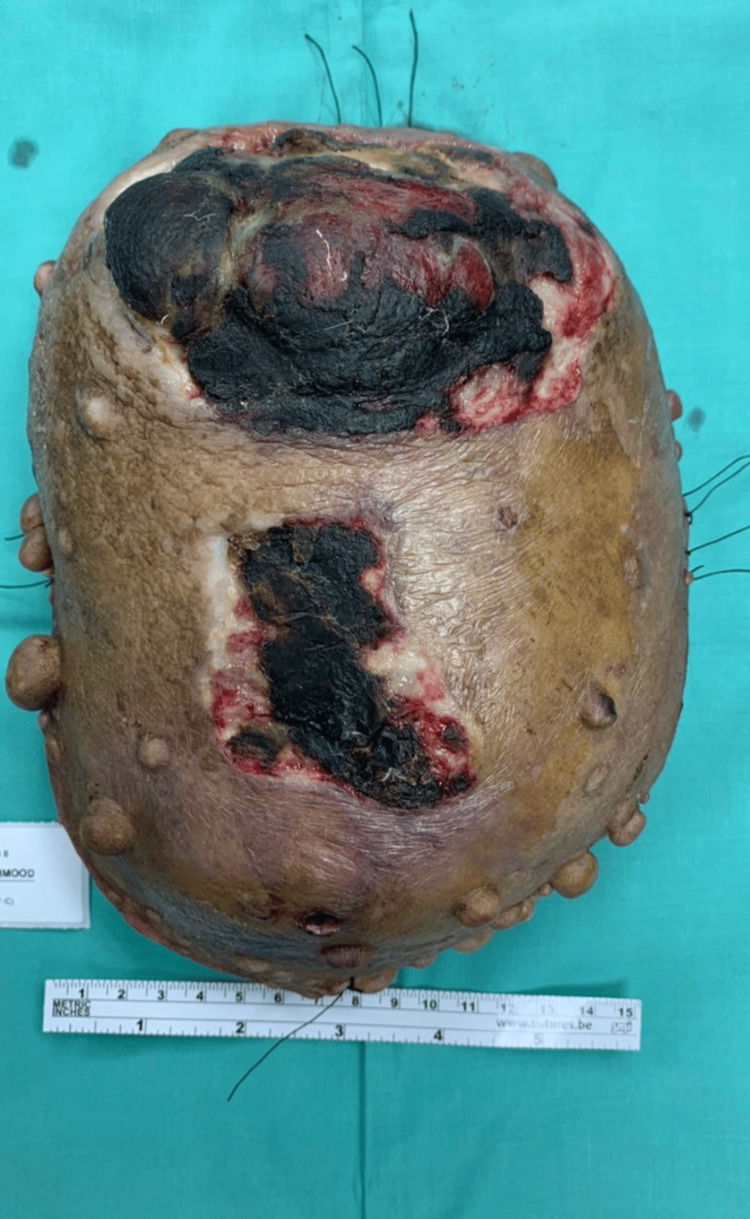
Tumor excised (view from the apex of the mass).

**Figure 4:**
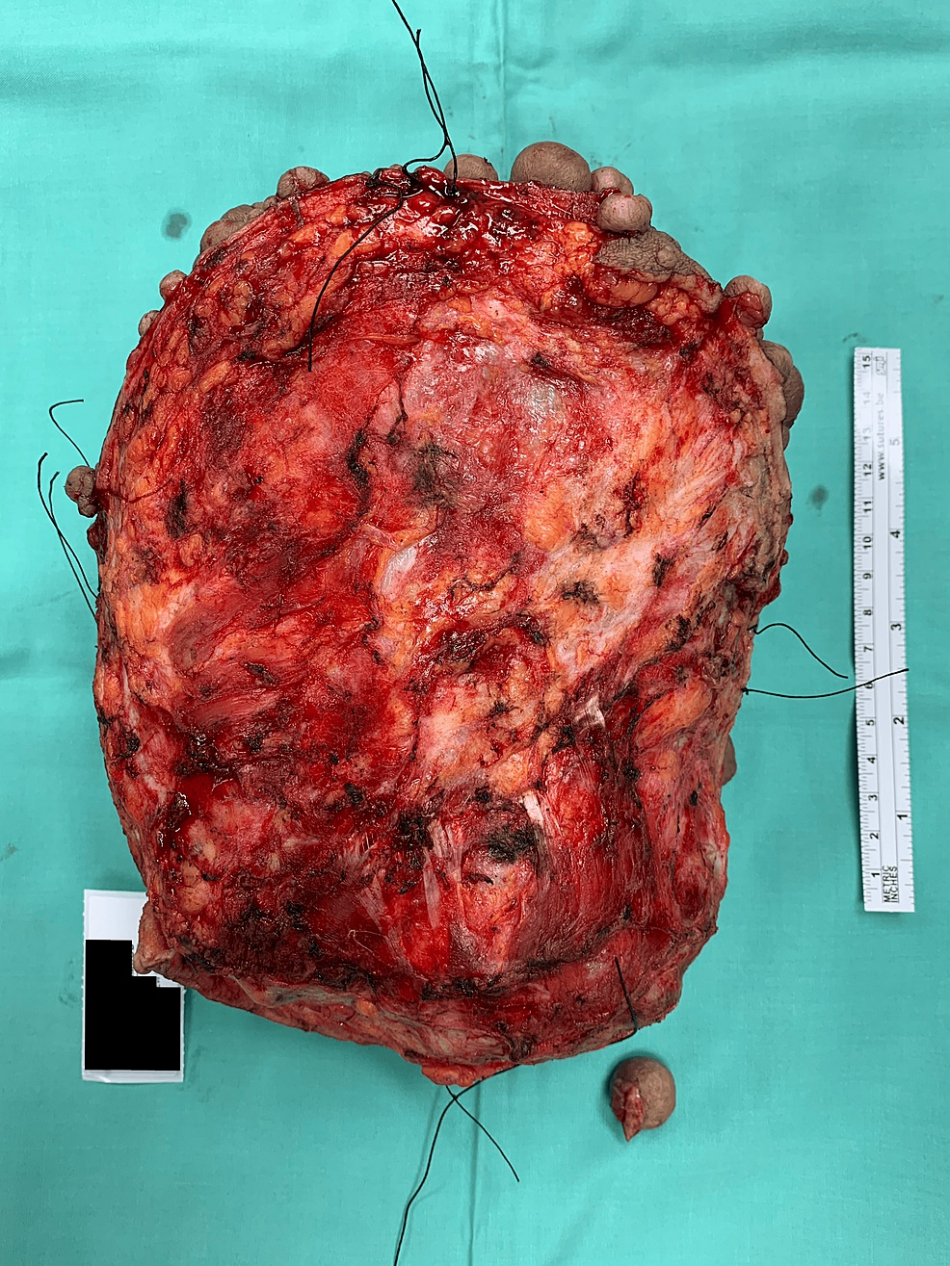
Mass excised (view from the base of the mass).

**Figure 5:**
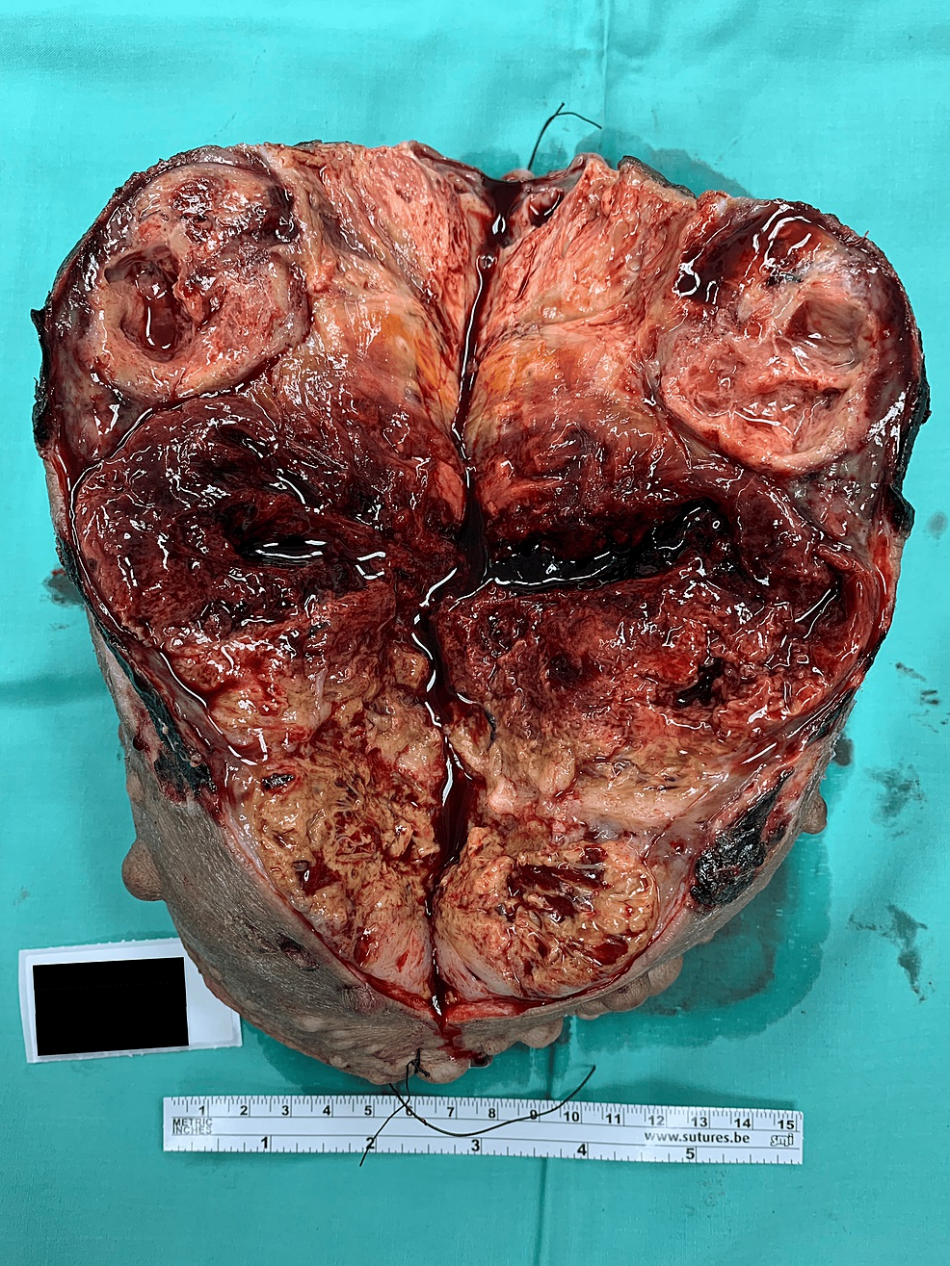
The appearance of the mass after cutting it open.

**Figure 6:**
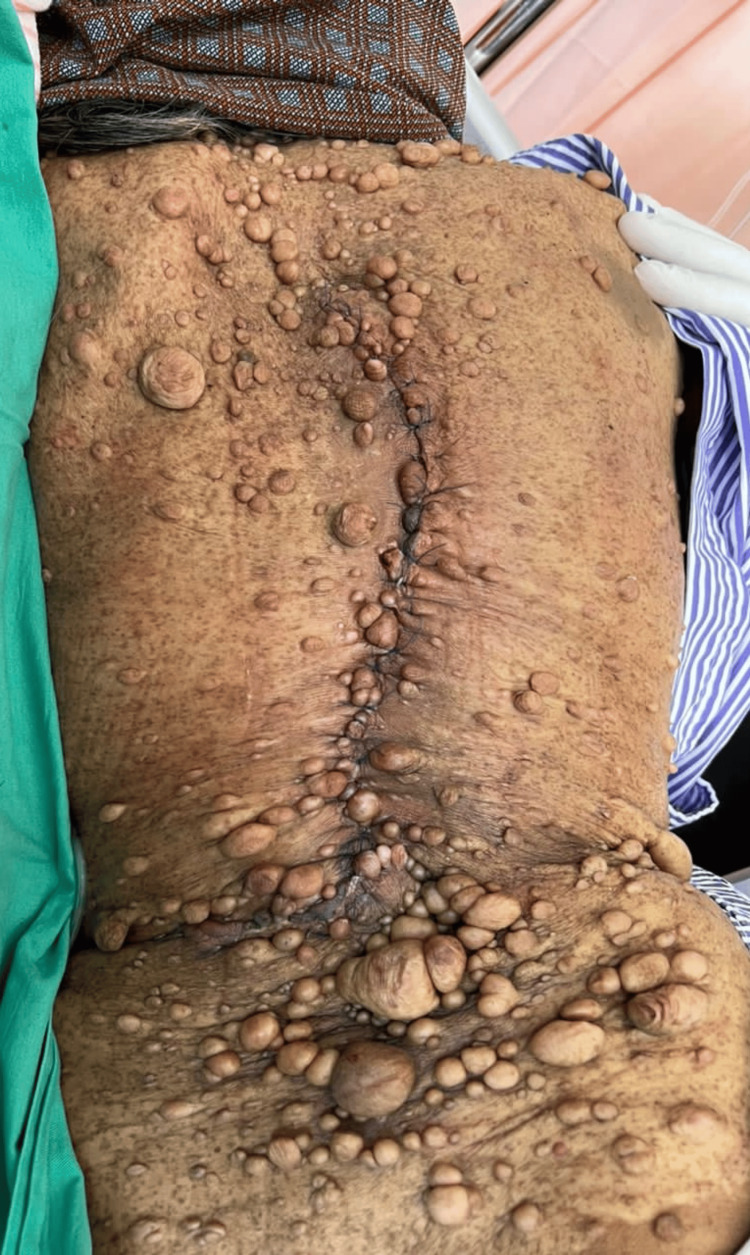
Clinical picture of the patient's back two weeks after surgery.

A 64-year-old lady with underlying neurofibromatosis presented with back swelling for the past five years. The swelling was painless, and it had rapidly increased in size for the past year with ulceration. She denied any fever; however, the ulcer was foul-smelling and associated with on-and-off pus discharge. However, she sought treatment for this problem multiple times and received antibiotics. She was treated for infected neurofibromatosis by a general practitioner. Due to an unresolved infection, she was referred to a tertiary center for further management. Upon review by the orthopedic team, there was a huge mass on her back with necrotic skin (Figures 1-2). The surrounding skin was erythema, and it was hard in consistency. She underwent a wide local excision of the mass. The intraoperative finding was an ulcerated soft tissue tumor measuring about 15 cm x 20 cm at the posterior trunk with central hemorrhagic necrosis (Figures 3-5). The mass is attached to the fascia and some parts of the erector spinae muscle. The mass was sent for histopathology examination (HPE). The HPE came back with a malignant peripheral nerve sheath tumor. Computed tomography (CT) scan of the thoracic, abdominal, and pelvis (TAP) showed multiple lung nodules and lytic bone lesions. She underwent adjuvant radiotherapy after surgery. She was doing well after surgery, as she was able to lie flat. Figure 6 shows her back condition two weeks after surgery. After six months of serial follow-up in the clinic, there was no sign of recurrent tumors.

## Discussion

Neurofibromatosis is a rare genetic disorder inherited in an autosomal dominant pattern. It typically causes benign nerve tumors and growth in other parts of the body. There are three types: NF1, NF2, and schwannomatosis (SWN). NF1 is characterized by multiple cutaneous neurofibromas, cafe'-au-lait spots, plexiform tumors, Lish nodules, axillary or inguinal freckling, and optic gliomas. The disease might emerge in any part of the system. It poses a risk of malignant transformation to certain cancers, such as MPNSTs, brain tumors, and breast cancer, which are much more common. 

MPNSTs, also known as neurofibrosarcoma or malignant schwannoma, are malignant tumors that may occur as solitary lesions or be associated with neurofibromatosis. Most of the cases are associated with NF1. It was found that patients with NF1 had a 4.6% risk of MPNST [2]. The incidence of MPNST among the general population is 0.001%. Thus, the risk for the development of MPNST appears to be 4,600 times greater in patients with NF1 than in the general population [2]. NF1-associated MPNSTs typically begin as PNs. PN is an irregular, thick, and uncircumscribed tumor of the peripheral nerve sheath. PN is considered a precancerous lesion that can progress to malignant transformation.

In clinical manifestation, it is difficult to distinguish PN from MPNST since they both exhibit similar appearances. Some patients with PN remain asymptomatic throughout their lives. A history of rapid growth in a prior stable neurofibroma is suspicious of malignant transformation. Any masses greater than 5 cm carry a high risk of being malignant [6]. A proper evaluation should be made if we encounter masses that are greater than 5 cm. The most important action is to rule out malignant tumors, which is the most appropriate action. Delayed diagnosis may lead to invasion of adjacent structures and distant metastasis. In addition, ulcerated, huge, and surrounding erythematous appearances are signs of malignant transformation of soft tissue tumors. In this case, the patient presented with a foul-smelling discharge and ulceration surrounding the mass. She was treated for infected neurofibromatosis, causing delays in treatment. There are no such cases of infected neurofibromatosis, however, there is one case reported in the literature with infected neurofibroma that improved clinically with a 10-day course of antibiotics [7].

The behavior of MPNST is badly aggressive. The main treatment for MPNST is surgical resection, while the effect of adjuvant therapies on prognosis remains unknown [8,9]. The only effective treatment is a complete surgical resection to achieve negative margins. Adequate margin is critical to controlling local recurrence and ultimately patient survival. Complete resection results in a lower recurrence and a higher five-year survival rate than those with positive margins [9,10]. The prognosis of patients with MPNST is currently reported to be various in numerous studies, but the five-year survival of patients remains poor in general [8]. NF1 status has been reported to be a risk factor for the prognosis of MPNST [11-13].

## Conclusions

MPNST is mostly associated with NF1. Most PNs progress to malignant lesions. Surgeons should rule out the possibility of malignant transformation when dealing with a large tumor. Early intervention can help prevent problems and malignant changes. Appropriate surveillance in patients with NF1 may control malignant PN transformation and improve survival. Despite the existence of only one reported case of infected neurofibromatosis, it is crucial to rule out malignant transformation before treating the infection. The main treatment is surgical resection with a clear margin; however, most of the studies showed that survival and local recurrence are poor.
